# Optimal clinical protocols for total-body 18F-FDG PET/CT examination under different activity administration plans

**DOI:** 10.1186/s40658-023-00533-y

**Published:** 2023-02-18

**Authors:** Yanchao Huang, Meng Wang, Li Jiang, Lijuan Wang, Li Chen, Qiaoyu Wang, Jiatai Feng, Jingyi Wang, Wanbang Xu, Hubing Wu, Yanjiang Han

**Affiliations:** 1 Laboratory for Quality Control and Evaluation of Radiopharmaceuticals, Department of Nuclear Medicine, Nanfang Hospital, Southern Medical University, Guangzhou, China; 2 Central Research Institute, United Imaging Healthcare, Shanghai, China; 3 Department of Traditional Chinese Medicine, Guangdong Institute for Drug Control, Guangzhou, China

**Keywords:** Total-body PET/CT, ^18^F-FDG, Protocol optimization, Fast scanning, Low-dose

## Abstract

**Background:**

Highly sensitive digital total-body PET/CT scanners (uEXPLORER) have great potential for clinical applications and fundamental research. Given their increasing sensitivity, low-dose scanning or snapshot imaging is now possible in clinics. However, a standardized total-body ^18^F-FDG PET/CT protocol is still lacking. Establishing a standard clinical protocol for total-body 18F-FDG PET/CT examination under different activity administration plans can help provide a theoretical reference for nuclear radiologists.

**Methods:**

The NEMA image quality (IQ) phantom was used to evaluate the biases of various total-body ^18^F-FDG PET/CT protocols related to the administered activity, scan duration, and iterations. Several objective metrics, including contrast recovery (CR), background variability (BV), and contrast-to-noise ratio (CNR), were measured from different protocols. In line with the European Association of Nuclear Medicine Research Ltd. (EARL) guidelines, optimized protocols were suggested and evaluated for total-body ^18^F-FDG PET/CT imaging for three different injected activities.

**Results:**

Our NEMA IQ phantom evaluation resulted in total-body PET/CT images with excellent contrast and low noise, suggesting great potential for reducing administered activity or shortening the scan duration. Different to the iteration number, prolonging the scan duration was the first choice for achieving higher image quality regardless of the activity administered. In light of image quality, tolerance of oncological patients, and the risk of ionizing radiation damage, the 3-min acquisition and 2-iteration (CNR = 7.54), 10-min acquisition and 3-iteration (CNR = 7.01), and 10-min acquisition and 2-iteration (CNR = 5.49) protocols were recommended for full-dose (3.70 MBq/kg), half-dose (1.95 MBq/kg), and quarter-dose (0.98 MBq/kg) activity injection schemes, respectively. Those protocols were applied in clinical practices, and no significant differences were observed for the SUV_max_ of large/small lesions or the SUV_mean_ of different healthy organs/tissues.

**Conclusion:**

These findings support that digital total-body PET/CT scanners can generate PET images with a high CNR and low-noise background, even with a short acquisition time and low administered activity. The proposed protocols for different administered activities were determined to be valid for clinical examination and can maximize the value of this imaging type.

**Supplementary Information:**

The online version contains supplementary material available at 10.1186/s40658-023-00533-y.

## Background

Positron emission tomography combined with computed tomography (PET/CT) is a powerful modality to noninvasively collect information about the biodistribution of radiotracers in organs/tissues and anatomic structures in vivo [1–4]. This imaging modality shows enormous potential for various clinical applications including oncology [5, 6], cardiology [7, 8], and neurology [9]. ^18^F-fluoro-deoxyglucose (^18^F-FDG, an analog of glucose) is a common radiotracer widely used in routine oncological PET/CT examinations. Aggressive tumors commonly show a glucose-avid high metabolic feature termed the Warburg effect [10]. ^18^F-FDG PET/CT examination thus plays a crucial role in tumor localization [11], tumor staging [12, 13], and monitoring treatment responses [14–16], with diagnostic accuracy intimately depending on PET images.

PET image quality is greatly restricted by the performance of the PET/CT scanner and reconstruction algorithm, which directly determines whether it is necessary to increase or decrease the injection activity and acquisition time [17]. Over the past two decades, commercial PET/CT scanner detectors have transitioned from using a combination of bismuth germanate oxide scintillators, and photomultiplier tubes (BGO-PMTs) to digital mode—lutetium-based scintillators read out by silicon photomultipliers (SiPMs) [18–20]. This evolution has led to huge improvement in detector detection efficiency and made the application of time-of-flight (TOF) technology possible [21, 22]. uEXPLORER (United Imaging Healthcare, Shanghai, China), a model of total-body digital PET/CT scanner, utilizes SiPMs and tiles them along the axial field-of-view (AFOV) to 194 cm [23]. This design enables entire human body PET coverage in one-bed scan and increases the effective detection angle, thereby improving the sensitivity of the total PET system [24–26]. With this improved sensitivity (40-fold gain) [27], both the acquisition time and administered activity can be dramatically reduced for PET scans [28] while maintaining image quality. These are favorable in terms of both the economic aspect and patient radiation dose management.

The uEXPLORER has used in clinical practice since 2018 and is now used worldwide. Several prior studies have reported advantages of digital PET/CT scans with a long axial field-of-view in clinical practice, including the potential of low doses [29, 30] and snapshot PET/CT imaging in adults [31], fast scanning for pediatric patients [32], small lesion localization [33, 34], etc. Despite that, how to effectively use total-body PET/CT scanners to meet the ultra-high throughput of patients at PET centers in China is still unclear. Therefore, it is of great importance to unify the scanning protocol to achieve repeatability and reproducibility in total-body ^18^F-FDG PET scans.

At present, the protocol of total-body PET/CT scanners is adjusted to meet the demands of a highly diagnostic performance and to maximize patient throughput in clinical practice. To our knowledge, a protocol for total-body PET/CT (with improved sensitivity) to meet different imaging conditions has not yet been proposed. To present a standard procedure for static ^18^F-FDG PET/CT imaging examination, EARL’s guideline [11] detailed patient preparation and precautions, recommendations for ^18^F-FDG administered activity, and data acquisition protocols. In the present study, we determine the optimal administered activity and reconstruction setting (including scanning duration and iteration numbers) by evaluating the contrast recovery (CR) and noise properties of NEMA IQ phantom images [35, 36] and then verify the optimized protocol in total-body PET/CT clinical examinations.

## Materials and methods

### PET/CT system

The PET component of the uEXPLORER PET/CT device comprises 8 PET units (24.02 cm axial length) along the axial direction, with the units arranged cylindrically in a ring with a 78.6 cm diameter (detector face-to-face). Each unit consists of 24 detector modules, each of which contains an array of 70 block detectors. Each detector block is composed of a 7 × 6 array of lutetium-yttrium oxyorthosilicate (LYSO) crystals coupled to four SiPMs (SensL J-series). In total, the PET part of the uEXPLORER comprises 13,440 detector blocks with a total of 564,480 LYSO crystals and 53,760 SiPM channels. The energy window of the scanner is 430–645 keV, while the time-of-flight (TOF) resolution of the system is ~ 430 ps. The coincidence time window ranges from 4.5–6.9 ns varies with different PET units.

### NEMA IQ phantom preparation

All measurements were performed and evaluated with respect to the NEMA NU2-2007 manual [37]. The NEMA IQ phantom has a background compartment with a measured volume of 9.8 L, a low-density lung insert in the center, and 6 fillable spheres (with diameters of 10, 13, 17, 22, 28, and 37 mm) arranged circumferentially around the lung insert. The background compartment of the phantom was filled with ^18^F-FDG solution with an activity concentration of ~ 5.2 MBq/L. The 37 and 28 mm cold spheres were filled with water, while the four remaining smaller hot spheres were filled with ^18^F-FDG solution with an activity concentration four-fold denser than the background concentration. To obtain PET images derived using different administered activities, PET examination of NEMA IQ phantom can be performed at the same bed position on several timepoints to cross few half-lives of ^18^F radionuclide. Based on the decay rate equation, the injected activity can be estimated by the instantaneous activity concentration multiplied by a decay correction factor of 1.460. Thus, a series of injected activities was estimated, e.g., 7.61 MBq/L (0.206 mCi/kg) for the original measurement, 3.81 MBq/L (0.103 mCi/kg, group full-dose) after the first half-life, 1.90 MBq/L (0.051 mCi/kg, group half-dose) after the second half-life, 0.95 MBq/L (0.026 mCi/kg, group quarter-dose) after the third half-life, 0.48 MBq/L (0.013 mCi/kg, group 1/8-dose) after the fourth half-life, 0.24 MBq/L (0.006 mCi/kg, group 1/16-dose) after fifth half-life, and 0.12 MBq/L (0.003 mCi/kg, group 1/32-dose) after the sixth half-life.

### Phantom measurements

NEMA IQ phantom measurement was performed at the center of the PET system, as the axial sensitivity profiles of the uEXPLORER showed a peak sensitivity (up to 147 kcps/MBq) plateau ± 48.5 cm from the center [38]. All necessary corrections were performed, e.g., attenuation and scatter correction. Before PET measurement, a low-dose CT scan (100 kV tube voltage, 10 mAs exposure, 1.0125 pitch) was performed for CT-based attenuation correction (CTAC). To mimic the conditions of clinical practice, all images were reconstructed using the list-mode ordered-subsets expectation maximization algorithm incorporating TOF and point-spread function modeling (OSEM-TOF-PSF) in which several reconstruction parameter settings are fixed, e.g., FOV = 600 mm with a Gaussian post-filter (full width at half maximum = 3 mm), matrix 192 × 192 (3.125 × 3.125 mm^2^), and 2.89 mm slice thickness. For the different injected doses, the corresponding PET data of NEMA IQ were reconstructed into a series of images (e.g., 40 s, 1 min, 2 min, 3 min, 5 min, 10 min and 30 min) by truncating the acquisition duration. Additionally, we tuned the iteration number from 2 to 15 to explore the effect of iteration convergence on image quality.

### Image analysis and interpretation

Using software developed in-house that runs on MATLAB (MathWorks, MA, USA), regions of interest (ROI) were automatically drawn to include the spheres and background of the phantom image according to NEMA NU2-2007 criteria. To optimize the total-body ^18^F-FDG PET/CT scanning protocol, the background variability (BV) and CR were considered the measures of image noise and quantitation accuracy, respectively. BV was defined by the standard deviation (SD) of 12 ROIs located at five slices (a total of 60 background ROIs of each size)—one central slice crossing the sphere center and four close slices at ± 1 cm and ± 2 cm—divided by the mean activity of these 60 background ROIs. CR was defined as follows [39]:

1 $${\text{CR}} = \frac{{{\raise0.7ex\hbox{${M_{{\text{H}}} }$} \!\mathord{\left/ {\vphantom {{M_{{\text{H}}} } {M_{{\text{B}}} }}}\right.\kern-0pt} \!\lower0.7ex\hbox{${M_{{\text{B}}} }$}} - 1}}{{{\raise0.7ex\hbox{${C_{{\text{H}}} }$} \!\mathord{\left/ {\vphantom {{C_{{\text{H}}} } {C_{{\text{B}}} }}}\right.\kern-0pt} \!\lower0.7ex\hbox{${C_{{\text{B}}} }$}} - 1}} \times 100\% ,$$

where *M* is the count from the PET image and *C* is the activity in hot spheres and background ROIs (subscripts H and B, respectively). Notably, the contrast-to-noise ratio (CNR), which is considered a key measure of PET imaging performance, is defined as CR divided by BV. First, we regrouped the subsets based on the BV value, where the upper threshold of BV was 15% [40]. We then calculated the CR in each filtered condition and produced the CNR that provides a quantitative measure of balance for both BV and RC with the same weight ratio [41, 42]. A subjective method of assessment—namely the 5-point Likert scale—was also used to evaluate overall impression of image quality and image noise. All of the center slice images were independently rated by two nuclear radiologists (a senior radiologist with > 5-year experience and a junior radiologist with 1 year of experience reading PET/CT scans) in blinded. The 5-point Likert scale of overall image quality comprises five categories: (1) poor, (2) barely diagnostic, (3) clinically acceptable, (4) superior to the regular quality of daily practice, and (5) excellent. The relationship plot between the CNR value and 5-point Likert scale combines objective and subjective assessment for optimal protocol recommendation. Specifically, the protocol that generates images with high quality (e.g., a score greater than 4- on the 5-point Likert scale) and simpler acquisition settings (e.g., a shorter scan duration and lesser iterations) was preferred in this study.

To facilitate the localization of small lesions, our results—such as BV, CR, and CNR—are mainly demonstrated for hot-spheres with a diameter of 10 mm.

### Clinical PET/CT imaging

A total of 15 oncological patients were enrolled in this prospective study. Patient characteristics are listed in Table 1. The exclusion criteria included exercise in the prior 24 h; history of diabetes; and inability to lie supine and still inside the scanner for the acquisition duration. The patients were divided into three groups of three dose levels (full-dose, half-dose, and quarter-dose); each group included five patients. To avoid imaging failure, the lower injected dose levels were not evaluated. Patients in full-dose group, half-dose group, and quarter-dose group were injected with activities of 3.70 MBq/kg (0.1 mCi/kg), 1.85 MBq/kg (0.05 mCi/kg), and 0.93 MBq/kg (0.025 mCi/kg), respectively. All patients fasted for more than 6 h before the ^18^F-FDG PET examination. Total-body PET/CT examinations were performed with 10–15-min acquisition 60 min after intravenous injection of ^18^F-FDG. On the PET images, a spherical volume of interest (VOI) with a diameter of 2 cm was manually placed on the aortic arch, normal right liver lobe, muscle of left inner thigh, and normal lung tissue to calculate the organ/tissue SUV_mean_ value. Similarly, a size-adaptive VOI was manually placed on the major suspect lesion and one small metastatic lesion to calculate the lesion SUV_max_ value, respectively. Statistical comparisons were made using the paired t-test performed using the R statistical package. *p* values < 0.05 were considered statistically significant.

**Table 1:** Clinical characteristics of the enrolled oncological patients

Characteristics	Full dose (*n* = 5)	Half dose (*n* = 5)	Quarter dose (*n* = 5)
Age (years)	60.2 ± 8.2	53.2 ± 11.01	57.6 ± 7.96
Gender (F/M)	2/3	2/3	1/4
Height (cm)	160.6 ± 7.81	163.1 ± 4.34	164.2 ± 3.76
Weight (kg)	62.52 ± 4.72	63.88 ± 3.16	58.6 ± 5.33
BMI (kg/m^2^)	24.31 ± 2.00	24.08 ± 1.96	21.76 ± 2.05
Blood glucose level before injection (mmol/L)	6.5 ± 0.78	6.55 ± 0.15	6.13 ± 0.38
Injected dose (MBq)	255.37 ± 36.61	124.83 ± 16.29	65.71 ± 6.79
Histopathology			
Lung cancer	4	5	4
Colon cancer	0	0	1
Endometrial carcinoma	1	0	0

## Results

### NEMA IQ phantom

**Fig. 1:**
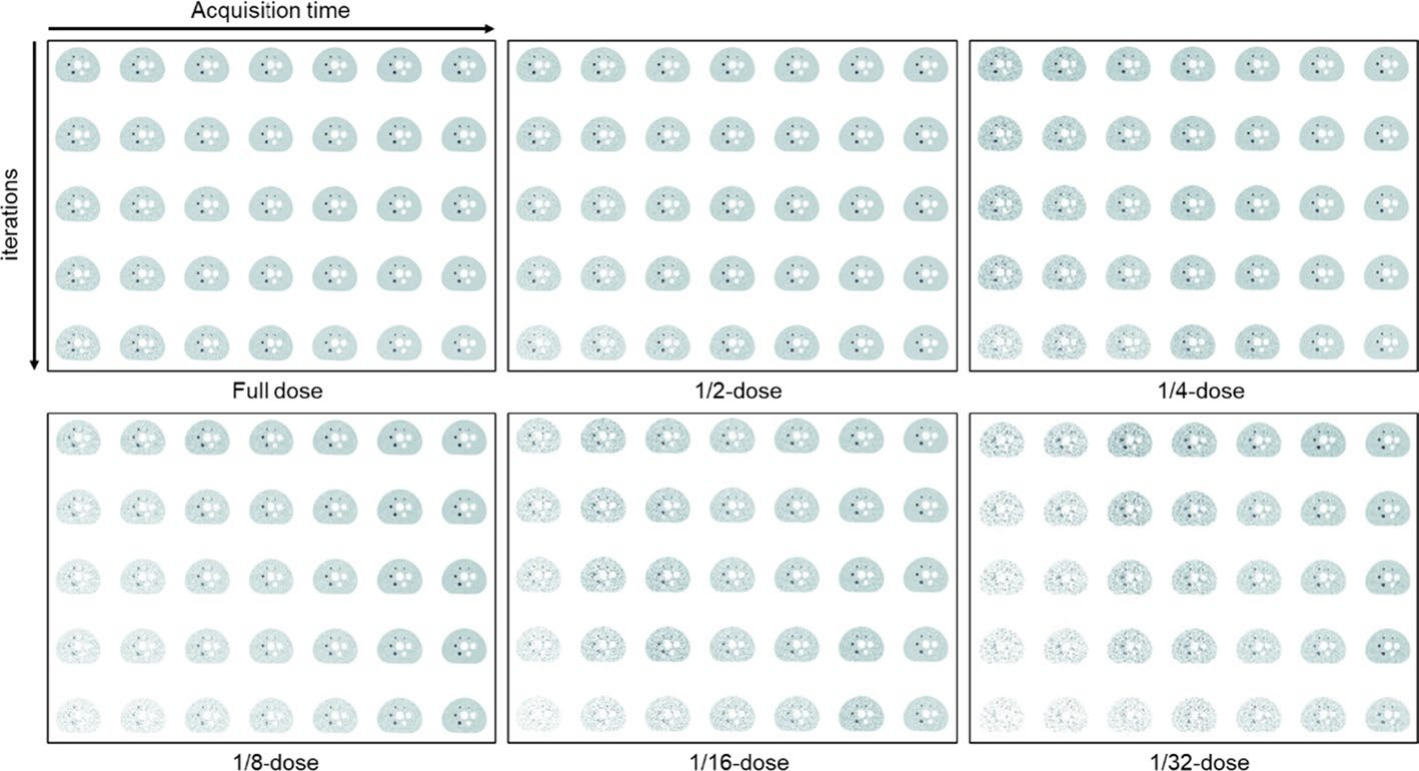
The schematic figure of transverse image slices of the NEMA IQ phantom achieved at the different acquisition time, iteration number, and injected activity. The PET raw-data were measured from the NEMA IQ phantom with a background activity of ~ 5.2 MBq/L (hot sphere-to-background ratio = 4:1) scanned for 30 min. By truncating the list-mode PET raw-data, a series of PET images were reconstructed into 40 s, 1 min, 2 min, 3 min, 5 min, 10 min and 30 min (ordered from left to right column), where the iteration number applied in OSEM-TOF-PSF reconstruction process was 2, 3, 4, 5, and 15 from top to bottom line, respectively. The activity per body weight was estimated from the instantaneous background activity concentration taking into account of the nature decay of ^18^F nuclide for a waiting time of 60 min after injection. The activity per body weight ranged from 0.24 to 7.61 MBq/L

Figure 1 depicts a duration-iteration-dependent PET image of the NEMA IQ phantom with different injected activities from the transverse view. Visually, the higher image quality requires a longer acquisition time, while the background noise increases with the increasing iteration number.

**Fig. 2:**
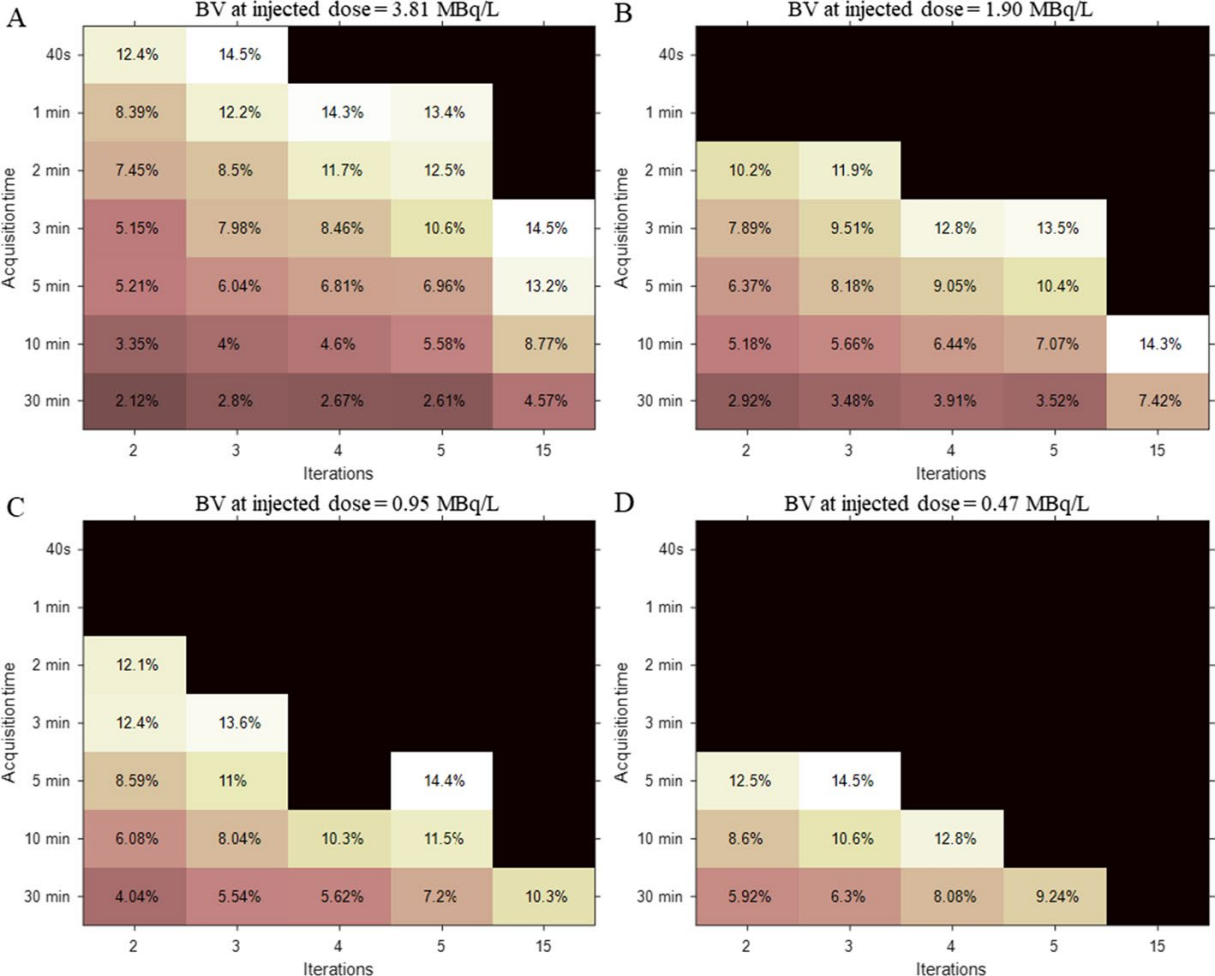
Background variability (BV) map, which is derived from NEMA IQ phantom measurement (hot sphere: background, 4:1) at four estimated doses, as a function of both factors—acquisition time and iterations. The dark area represents that BV value is more than 15%

Figure 2 illustrates the BV values at different injected activities from 0.47–3.81 MBq/L. As the number of iterations increases so does image noise, ultimately leading to a high BV value; this relationship is consistent with previously published reports [40]. For example, with a BV at injection activity of 3.81 MBq/L and 10-min acquisition duration, image noise increased at a factor of 2.66 compared with 2 and 15 iterations (3.35% at 2 iterations vs. 8.77% at 15 iterations). In contrast, a longer acquisition time results in minimum image noise. Specifically, extending the acquisition time from 10 to 30 min enables a reduction in BV from 8.77% to 4.57% for 15 iterations at 3.81 MBq/L. As the injected activity decreases, the BV value in the same condition increases, indicating worse image quality. However, the trend for the CR value (plotted in Additional file 1: Fig. 1) is quite different from the BV values presented. In general, an increase in iterations results in larger CR values. Such differences hinder the use of BV or CR values as independent indicators to evaluate image quality.

**Fig. 3:**
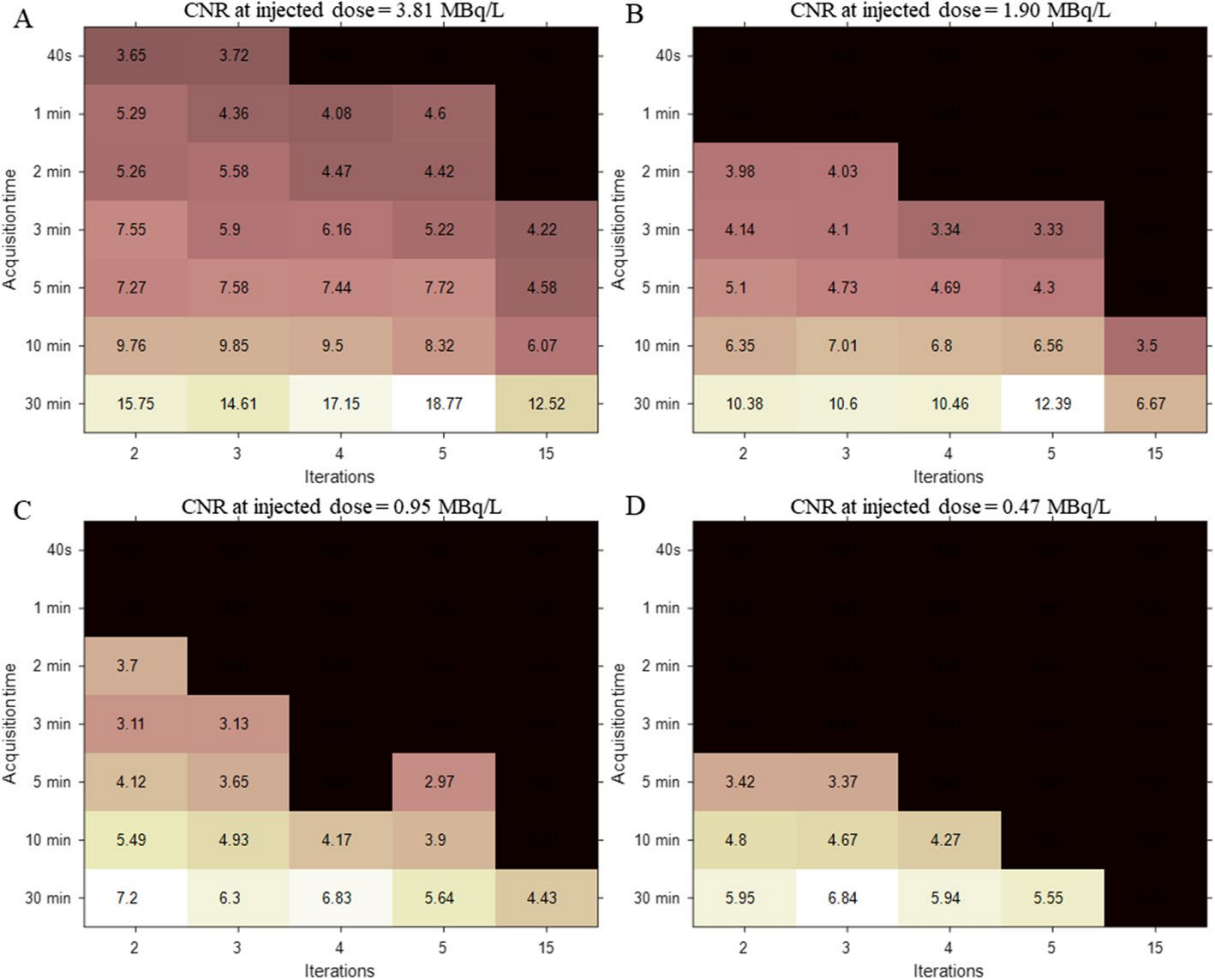
Contrast-to-noise ratio (CNR) map as a function of both factors—acquisition time and iterations at different estimated injected dose. The dark area represents the corresponding BV value that is more than 15%. The CNR values that excessed 5.0 were considered as acceptable for diagnosis

**Fig. 4:**
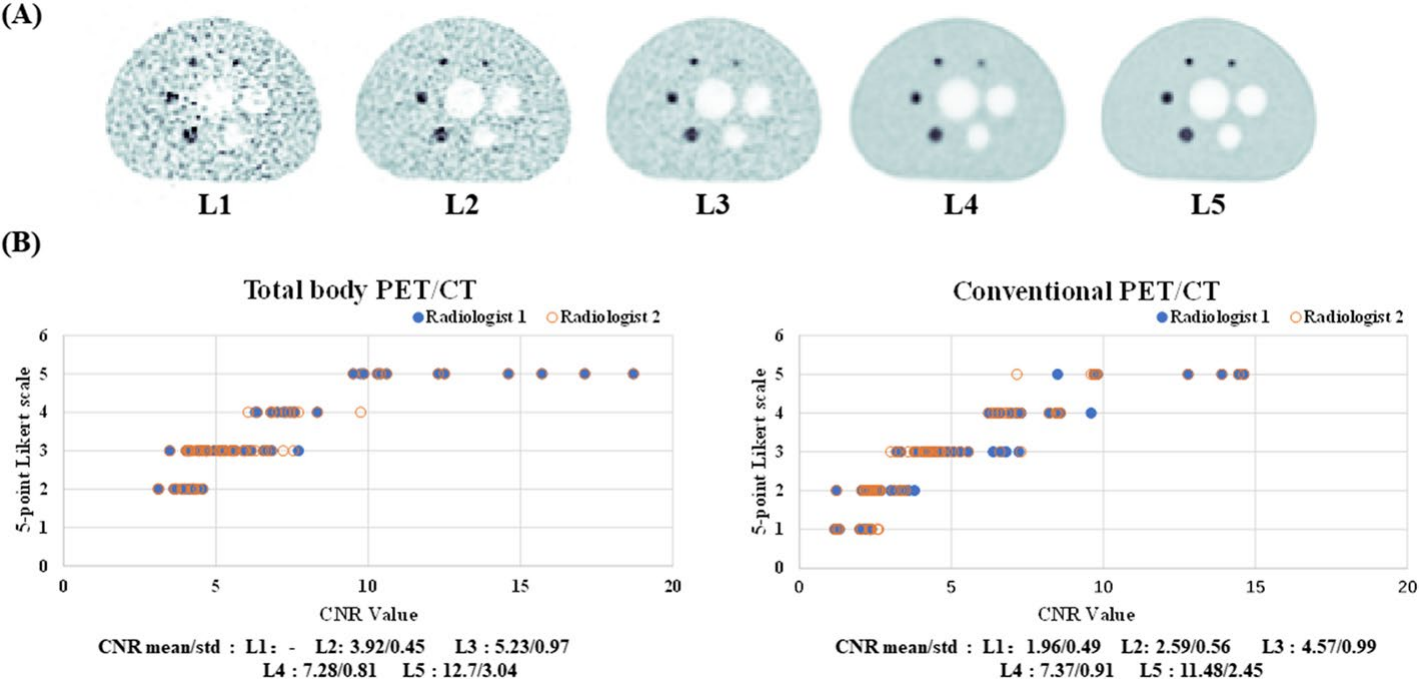
Relationship between the objective indicator (CNR) and the subjective assessment (5-point Likert scale). **A** The reference example images of grades 1–5 in the 5-point Likert scale (L1: poor, L5: excellent). **B** Scatter plot for the CNR value vs. 5-point Likert scale upon the total body PET/CT scanner and the conventional short AFOV PET/CT scanner (derived from Siemens Biograph mCT scanner in this study), respectively. The 5-point Likert scale was independently rated by two nuclear radiologists in blind

Figure 3 demonstrates an iteration-and-duration-dependent CNR for different injected activities while Fig. 4 presents the relationship between the objective image CNR value and subjective 5-point Likert scale score. In addition to the total body PET/CT scanner data, similar experimental results using a conventional short AFOV PET/CT scanner (Siemens Biograph mCT) are presented in Fig. 4 (the experimental setting and relative results are detailed in the Additional file 1). No substantial difference was found in the mean CNR value for each 5-point Likert scale grade between the long and short AFOV scanner. Hence, we intuitively equally exchanged these two indicators and defined a CNR of 5, 7, and larger than 9 as a grade of 3, 4, and 5 on the 5-point Likert scale for later analysis. Notably, grade 3 indicates clinical routine image quality using a conventional short AFOV digital PET/CT scanner, as described by Zhao et al. [32].

As shown in Fig. 3, the 30-min duration condition generally achieves higher CNR values than the other shorter durations; however, it is not recommended for clinical application considering patient tolerance and departmental throughput. For the injected activity of 3.81 MBq/L (full-dose), several protocols are promising (CNR > 7, i.e. 5-point Likert scale larger than grade 4) in different acquisition durations, e.g., 3 iterations for 10 min, 3 iterations for 5 min, and 2 iterations for 3 min. Accounting for the shortened scan duration and reconstruction time consumption, the 2 iterations for 3-min acquisition are more suitable for a full-dose scheme in routine clinical examination since it slightly degrades the image quality but accelerates data acquisition speed nearly one- to two-fold that for the 3 iterations for 5 min and 3 iterations for 10-min settings, respectively. As the injected activity decreased to a half-dose (1.90 MBq/L), the PET images of the phantom were similar quality (CNR = 7.01) to those for full-dose under the conditions of 3-iteration and 10-min scanning, and is the optimal protocol for the half-dose scheme. When the injected activity is 0.95 MBq/L (a quarter of a full-dose) the image degraded; in this case, the 10-min and 2-iteration acquisition were suggested, in which a maximal CNR of 5.49 was found. Notably, when the injected activity is 0.48 MBq/L (1/8 of a full-dose), the CNR could reach 5 in the protocol with 10 min and 2 iterations, implying the feasibility of a low-dose PET scan with total-body PET scanner.

### Examples of clinical images

The aforementioned protocols were verified and evaluated in each dose group and then compared with the standard uptake values (SUVs) for several organs/tissues.

**Fig. 5:**
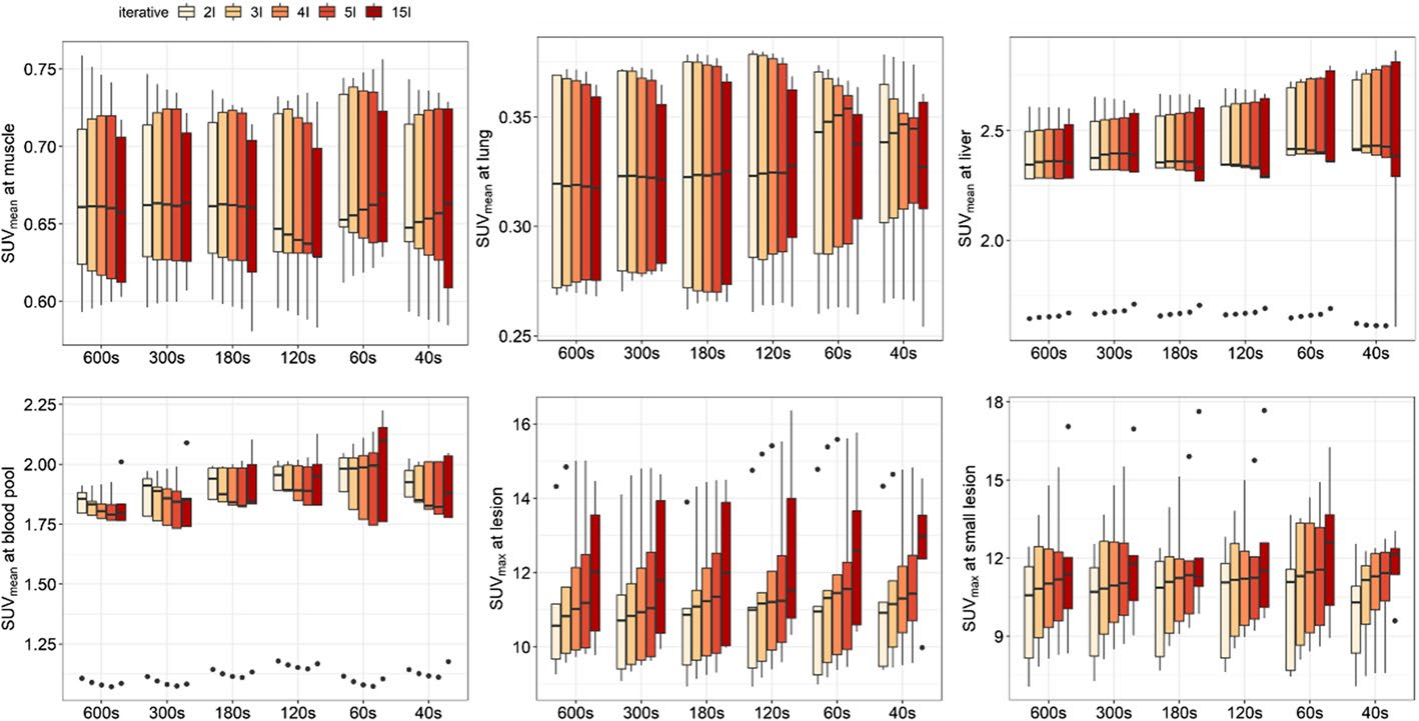
Iteration-dependent SUV_mean_ at muscle, lung, liver, and blood pool, and SUV_max_ at suspected major lesions and metastatic small lesions upon full-dose group for an acquisition time from 40 to 600 s, where the iterative numbers set at 2, 3, 4, 5, and 15

For the FDG injection with full dose, there was no significant difference for the mean SUV (SUV_mean_) in the blood pool (*p* > 0.1507), lung (*p* > 0.4206), muscle (*p* > 0.4206), and liver (*p* > 0.4206), showing robust stability that background did not change dramatically with the acquisition time and iterative number (Fig. 5). Although the median value increased as the iterative number increased in the intra-group comparison with the same acquisition time, the maximum SUV (SUV_max_) at suspected lesions and small lesions were not significantly different (all *p* > 0.2222). The similar behavior was observed in the groups with injected activity of half-dose and quarter-dose level (for further details, see Additional file 1: Fig. 2 and Fig. 3).

**Fig. 6:**
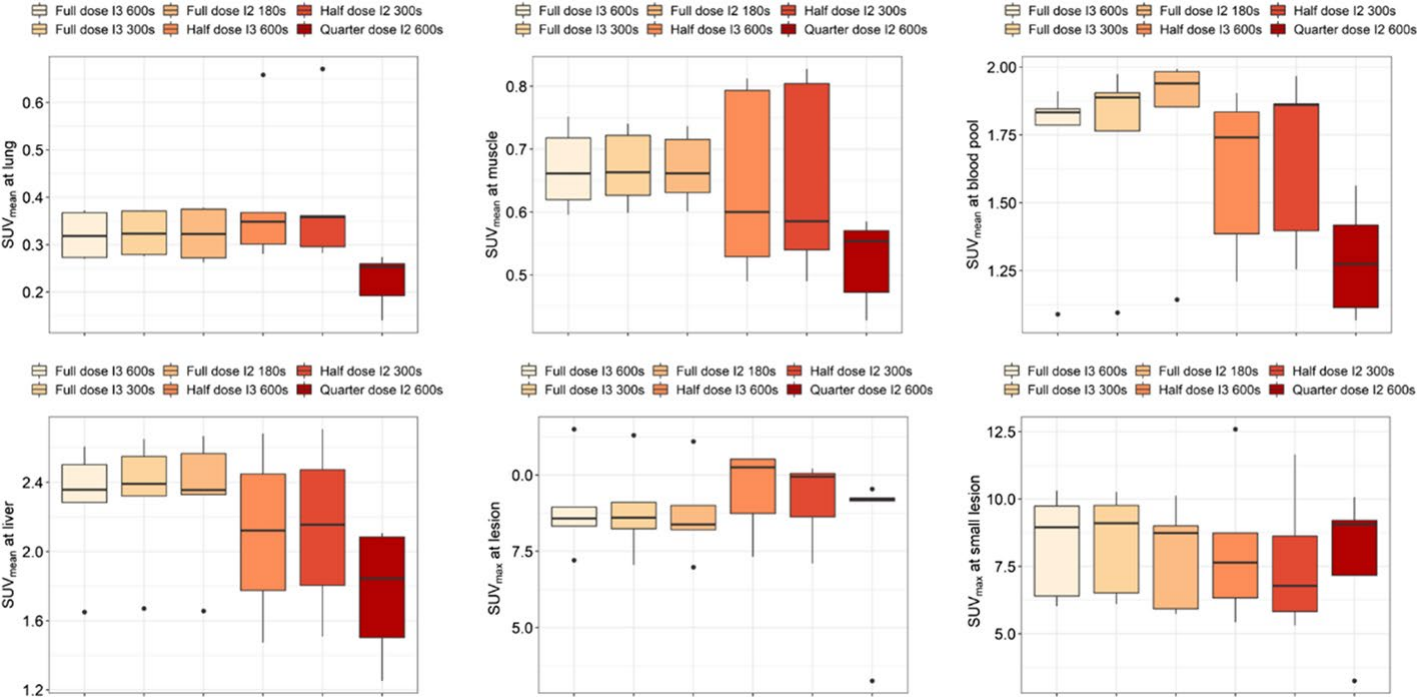
Comparison of SUV_mean_ at lung, muscle, blood pool and liver, and SUV_max_ at suspected major lesions and small metastatic lesions among six different suggested protocols that involves full-dose, half-dose, and quarter-dose

To clarify the clinical feasibility of low-dose protocols, we compared the SUVs of the blood pool, lung, muscle, liver, suspected major lesions, and small lesions derived from the aforementioned protocols (as shown in Fig. 6). Although the median SUV_mean_ values of lung, muscle, blood pool, and liver derived from the quarter-dose protocol with 600-s acquisition and two iterations were relatively lower than others, a significant difference was only observed in lung and muscle between the quarter-dose and full-dose (*p* < 0.0317). For other organs/tissues, the differences were not significant (all *p* > 0.0555). For more intuitive presentation, three clinical PET examination results with different injected activities (full-dose, half-dose, and quarter-dose) are shown in Additional file 1: Fig. 4, Fig. 5, and Fig. 6, respectively.

## Discussion

Total-body ^18^F-FDG PET/CT has the potential to provide fast acquisition with a low injected dose while assuring the PET images are of clinically acceptable quality. Based on NEMA IQ phantom studies, CNR can serve as a measure to quantitatively evaluate different PET/CT examination protocols. To achieve the best image quality using a total-body PET/CT scanner (uEXPLORER), several plausible protocols were proposed with three injected-activities schemes (0.925–3.7 MBq/kg, 0.025–0.1 mCi/kg) and successfully verified in the clinical practice.

The acquisition duration should be prolonged for images with a less noisy background. However, PET images with a long duration may be blurry because oncological patients are often in intense pain and tend to move during acquisition. The increased injected activity enables faster acquisition, thus avoiding motion artefacts that compromise image quality. However, it also results in a higher risk of ionizing radiation for patients. Therefore, achieving a balance between image quality, acquisition time, and injected activity is of great clinical significance. Although the full-dose (3.7 MBq/L, 0.1 mCi/L), 30-min acquisition, and 5-iteration scheme provided excellent image quality (CNR = 18.7), it is still not clinically feasible. Compared to a 20–30-min whole-body scan using a conventional short AFOV scanner, the full-dose scheme with 3-min acquisition using a total body scanner has tremendous clinical potential since it greatly increases patient throughput and achieves images with relative high quality (CNR > 7). Given the rapidly increasing sensitivity of uEXPLORER, it is able to further shorten the acquisition duration of the PET signal. A PET image, which is similar to an image derived from the conventional PET-CT scanner, has been found with reconstruction settings of 1 min and 2 iterations (CNR = 5.29, with a 5-point Likert scale larger than grade 3) using total-body PET/CT scanner. This fast scanning might improve the accuracy in pediatric diagnosis—eliminating the false-positive lesions caused by the motion, by only reconstructing with the data before the motion. Example of fast scanning eliminating the motion-induced false-positive lesions is shown in Additional file 1: Fig. 7. The results revealed that fast ^18^F-FDG PET/CT scans are feasible and useful for examining both adult and pediatric patients. Our findings are consistent with those of Li et al*.* and Tan et al. that fast-scan and low-dose schemes are helpful for adults with cancer pain [28, 43] and pediatric PET/CT examination under clinical settings [32].

To minimize radiation-induced DNA damage, the injected activity of ^18^F-FDG can be greatly reduced with total-body PET/CT scanners. This has been discussed by Shi et al. in previous studies [29, 30, 44], but the optimal protocols have not been systematically investigated. Based on the calculation of CNR, we designed several low-dose protocols to yield a PET image with clinically acceptable quality. The CNR calculated from ^18^F-FDG PET imaging with half-dose injection, 5-min acquisition, and 2 iterations was approximating similar to 5 (clinically acceptable image quality). Although the image is more blurred than that reconstructed using 10-min acquisition and 3 iterations (BV: 6.37% vs. 5.66%; CNR: 7.01 vs. 5.11), it met the diagnostic requirements while greatly enhancing patient throughput in our department. The injected activity can be further decreased to a quarter of the full-dose, whereas two iterations and more than 10-min acquisition time are suggested to generate a diagnosable image. Notably, the associated CNR is 5.49, which is slightly higher than that of the half-dose PET imaging with 5 min and 2 iterations. This relationship respects the rule of time-activity product. In clinical practice, oncological patients often have to undergo several PET examinations (interim PET) to monitor their treatment response. Such interim PET is especially important for immunotherapy, which may introduce side effects such as hyperprogression or pseudoprogression [45, 46]. With low-dose interim PET, the treatment response can be carefully evaluated between cycles so that adjustments to the treatment plan can be made accordingly and the patients can be protected from overexposure to radiation. In this context, the protocol of 1/8-dose (administered activity of 0.48 MBq/kg, 0.0125 mCi/kg, 10-min acquisition, 2-iteration) could be used in clinical practice with CNR > 5. However, the accuracy of SUV in the low-dose scheme should be further analyzed and evaluated using a large number of patients.

Reproducibility and repeatability are of great importance for PET multi-center studies [2, 47]. Given the injected activity of 3.7 MBq/kg (0.1 mCi/kg), our results indicate that with a reconstruction setting of 2 iterations and 1-min acquisition (CNR = 5.29, i.e., grade 3 on the 5-point Likert scale) for the OSEM algorithm, a total-body scanner generates images with similar quality and performance as conventional PET/CT scanner. This result agrees with Zhao et al*.*, who determined the equivalent state between uEXPLORER and other modern digital PET/CT scanners in terms of signal-to-noise ratio and tumor-to-background ratio [31]. For the half-dose, the PET images were reconstructed at two iterations with 5-min data. The half-dose images had a CNR of 5.11, which was consistent with images derived from the full-dose scheme using a total-body PET/CT scanner (CNR = 5.29). This half-dose protocol for the total-body scanner may generate equivalent images as a short AFOV (20–30 cm) PET/CT since they are at the same CNR level (~ 5). Furthermore, the images acquired with the total-body PET/CT scanner under the quarter-dose scheme had a similar CNR (CNR = 5.49) as the short AFOV scanner.

Based on the NEMA IQ phantom study, six protocols involving full-dose, half-dose, and quarter-dose methods were applied in clinical PET/CT examinations. Our results demonstrated that the SUV_mean_ at background and SUV_max_ at lesions were nearly robust. Nevertheless, SUV_mean_ at the lung and muscle exhibited a significant difference between the quarter-dose and full-dose protocols. However, this did not impede clinical diagnosis in our study.

There are some limitations of this study that should be noted. First, we should mention that the scatter and attenuation patterns, as well as the activity distribution of the NEMA phantom, are significantly different from patient scans due to differences in the body size. Such a translated result may induce biases in practical application; however, even for the quarter-dose clinical patient examination, there were no difficult diagnoses in our study. Besides, to avoid examination failure, ultra-low-dose conditions—such as 1/8 and 1/16 activity administered cases—were not performed in clinical patients. A larger standard human body-like phantom would be needed for further evaluation before guaranteeing clinical safety.

We should also mention that the aforementioned optimized protocols involved in this study merely adapt to the OSEM-TOF-PSF reconstruction algorithm for ^18^F-FDG PET/CT examinations. The application of different reconstruction algorithms, pixel sizes, and smoothing kernel are not considered. The optimal protocols for other radiotracers on total-body PET/CT scanners—such as ^68^ Ga-DOTA-TATE, ^68^ Ga-FAPI-04, and ^68^ Ga-PSMA—are not discussed in this study. Moreover, the involved cancer types were limited and the patient cohort was small. As a result, the results may be influenced by patient characteristics such as BMI, cancer type, age, etc.

## Conclusion

Utilizing a digital PET/CT scanner with a 194-cm-long axial field-of-view, total-body ^18^F-FDG PET images were generated using the standard protocol to yield high image quality. The optimal acquisition duration and iteration number for different injected activity conditions were recommended, which do help maximize the potential clinical applications of total body PET/CT scanners.

## Supplementary Information

**Additional file 1**. Supplementary file.

## Data Availability

Not applicable.
