# Supplementary material for: Optimal clinical protocols for total-body 18F-FDG PET/CT examination under different activity administration plans
Source: EJNMMI Phys. 2023 Feb 18;10:14. doi: 10.1186/s40658-023-00533-y (PMC9938848; doi:10.1186/s40658-023-00533-y)
Supplement: Supplementary file 1 — Additional file 1. Supplementary file. [file 40658_2023_533_MOESM1_ESM.docx]

# Supplementary


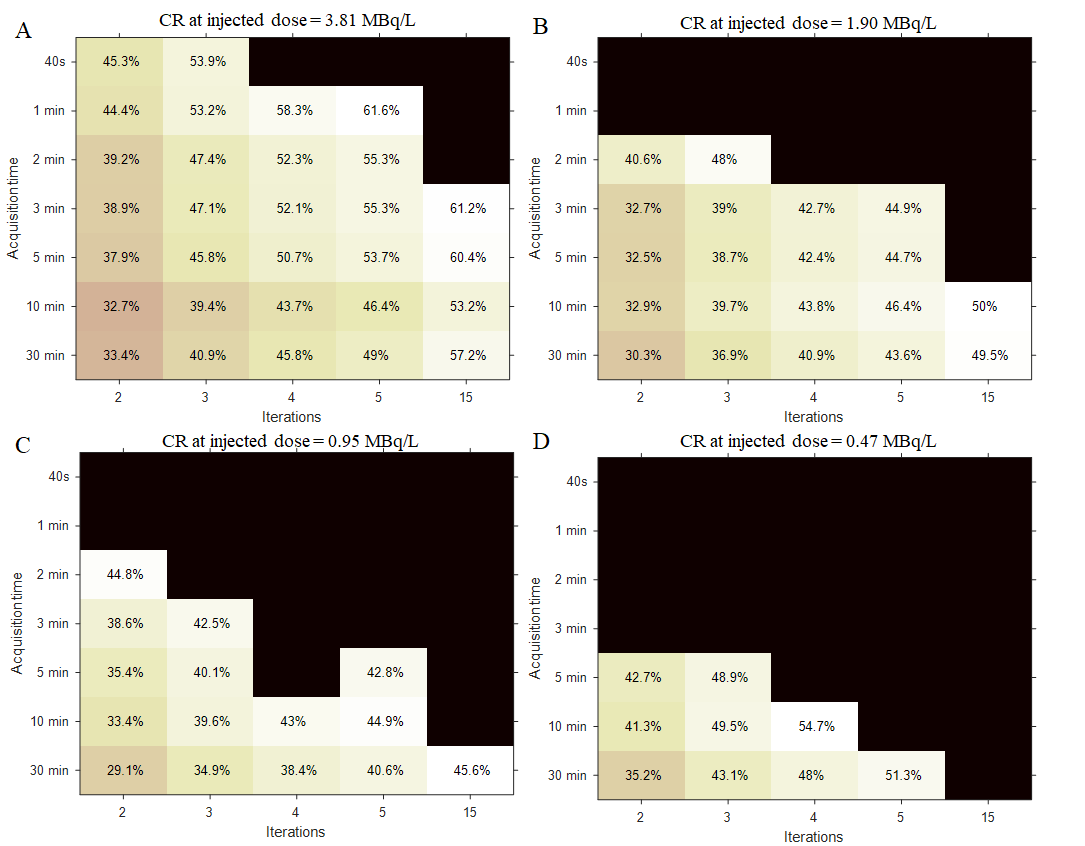


**Supplementary Figure 1.** Contrast recovery (CR) map, which is derived from NEMA IQ phantom measurement (hot sphere: background, 4:1) at four estimated doses that is injected in patients, as a function of both factors – acquisition time and iterations. The calculation of CR value is demonstrated for the hot-sphere with a diameter of 10-mm. Notably, the dark areas that represent BV values > 15% were excluded from this study.


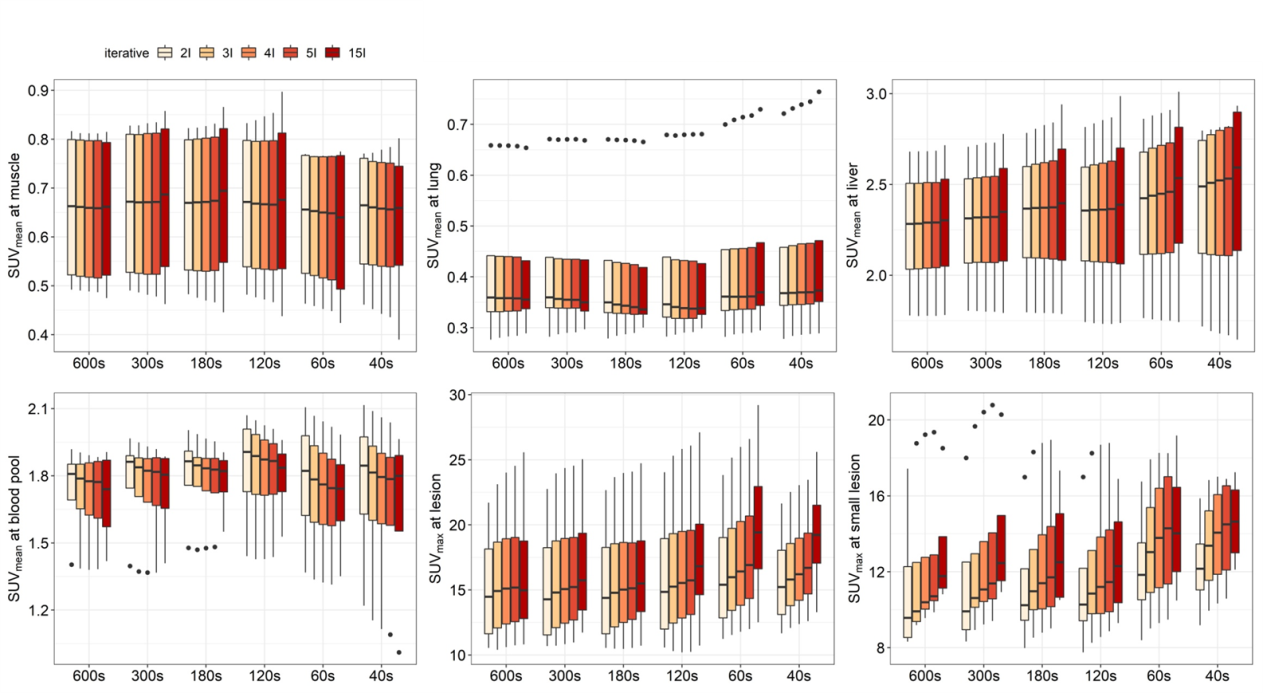


**Supplementary Figure 2.** Iteration-dependent SUV_mean_ at muscle, lung, liver, and blood pool, and SUV_max_ at suspected lesions and small lesions upon half-dose group for an acquisition time from 40 s to 600 s, where the iterative numbers set at 2, 3, 4, 5, and 15.


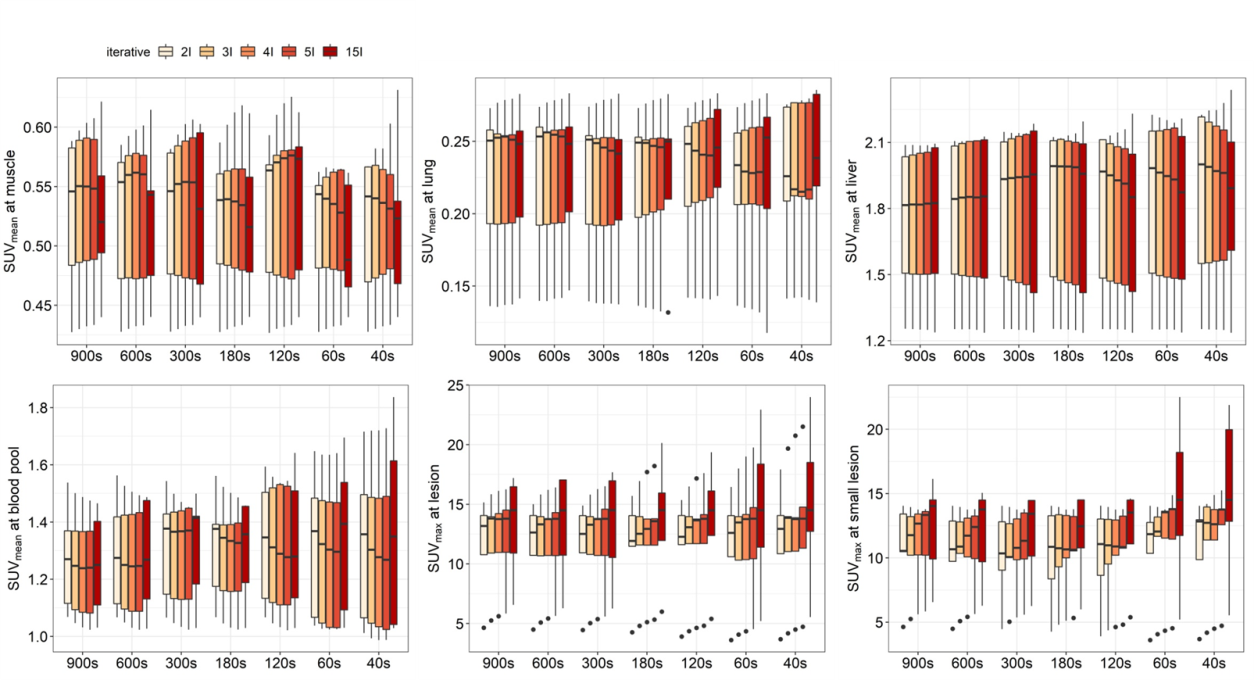


**Supplementary Figure 3.** Iteration-dependent SUV_mean_ at muscle, lung, liver, and blood pool, and SUV_max_ at suspected lesions and small lesions upon quarter-dose group for an acquisition time from 40 s to 900 s, where the iterative numbers set at 2, 3, 4, 5, and 15.

*Adult full-dose examination.* A 60-year-old male patient (65.0 kg, 167 cm) with advanced lung cancer was intravenously injected with 229.4 MBq ^18^F-FDG. A 10-min PET scan was performed on the uEXPLORER 64-min post-injection. Supplementary Fig. 4 demonstrated three PET images with different Likert grade, which have been reconstructed using the optimized protocols. The PET image acquired with 10-min duration and reconstructed with 3 iterations has better image quality. The PET images derived from the first 1- and 3-min were reconstructed with the same reconstruction parameters except the 2 iterations. Suspected small lesions (size range from 7.5-33.3 mm), including lymph nodes at the neck, pulmonary nodule, and metastasis, were easily localized on the images with 10-min acquisition, and these suspected lesions can also be identified even at 1-min PET images re-generated from the raw-data. In addition, the transverse image of the liver showed a relatively high homogeneous uptake even at 1-min PET image. Those results revealed that PET/CT examination using uEXPLORER with the optimal protocol (3-min acquisition time, 2 iterations and 3.7 MBq/kg) equally has a high detectability in the small lesion.

**Supplementary Figure 4.** A case of 64-year-old male patient with advanced lung cancer (65.0 kg, 167 cm) was intravenously injected with 229.4 MBq 18F-FDG (full-dose, 3.53 MBq/kg) and underwent a total-body PET examination at 64-min post-injection. Maximum intensity projection (MIP) images (A-C), transversal images crossing the lung (D-F) and the liver (G-I) were individually reconstructed with the acquisition time of the first 1-, 3-, and 10-min.

*1/2 full-dose reconstruction.* A 55-year-old male (60.3 kg, 170 cm) with lung cancer, who was intravenously injected with 109.89 MBq of ^18^F-FDG, underwent a 10-min PET scan at uEXPLORER after 60-min of injection. Supplementary Fig. 5 compares PET images reconstructed with two protocols (10 min and 3 iterations; 5 min and 2 iterations) using OSEM-TOF-PSF, 20 subsets, on a 3.125× 3.125×2.85 mm^3^ voxel grid. The boundary of cerebral cortex in 10-min and 3 iteration PET imaging is slightly clearer and the image contrast is better than that of 5-min and 2 iterations PET imaging. But the transverse images of other organs are visually similar between two protocols. Notably, the results of CNR indicate that the quality of 5-min and 2 iterations PET images with 1.85 MBq/kg (0.05 mCi/kg, 1/2 of full-dose) is similar comparing to conventional PET images with full-dose.


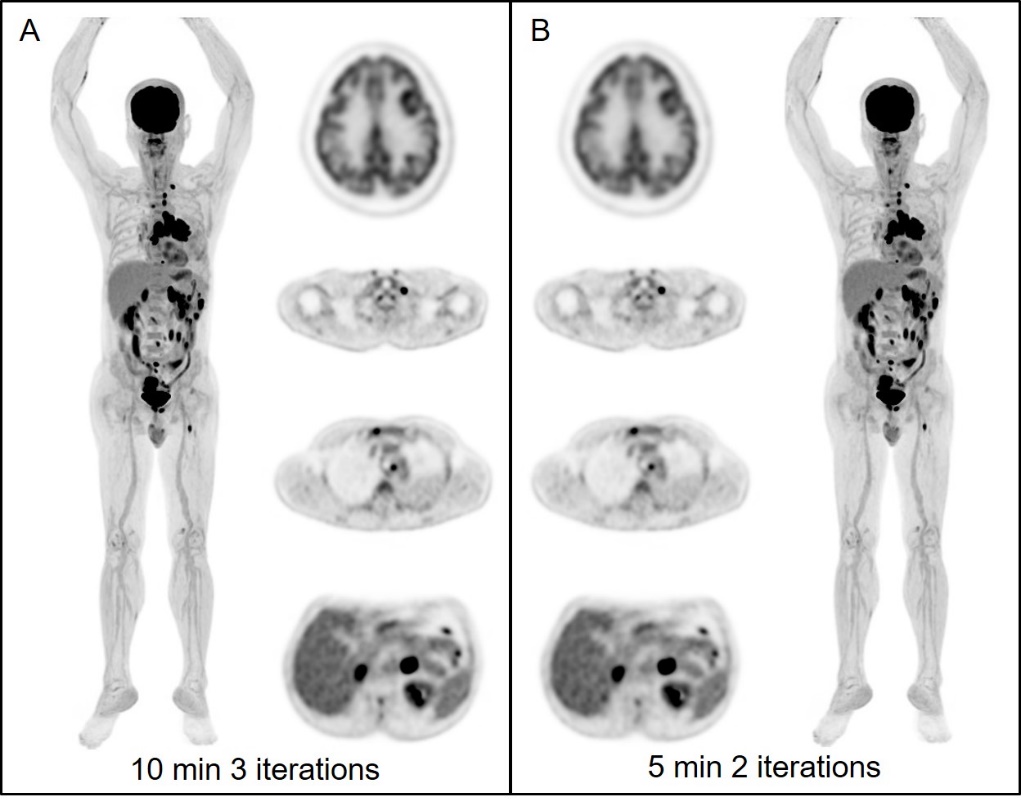
**Supplementary Figure 5.** A head-to-head comparison between two promising protocols of half-dose in a 55-year-old male patient (60.3 kg, 170 cm) with lung cancer. The PET/CT examination was performed at 60-min post-injection of 109.89 MBq ^18^F-FDG.

*1/4 full-dose reconstruction.* A 44-year-old female (53 kg, 156 cm) with colon cancer underwent a 10-min PET acquisition 63-min after an injection of 53.28 MBq ^18^F-FDG (1.01 MBq/kg, 0.027 mCi/kg). The PET images were reconstructed using OSEM-TOF-PSF with 20 subsets, 2 iterations on a 3.125×3.125×2.85 mm^3^ voxel grid (Supplementary Fig. 6). The uptake of liver showed great uniformity in the MIP. Even with 1/4 full-dose, the PET image could depict good organs/tissues boundaries with low image noise.

**
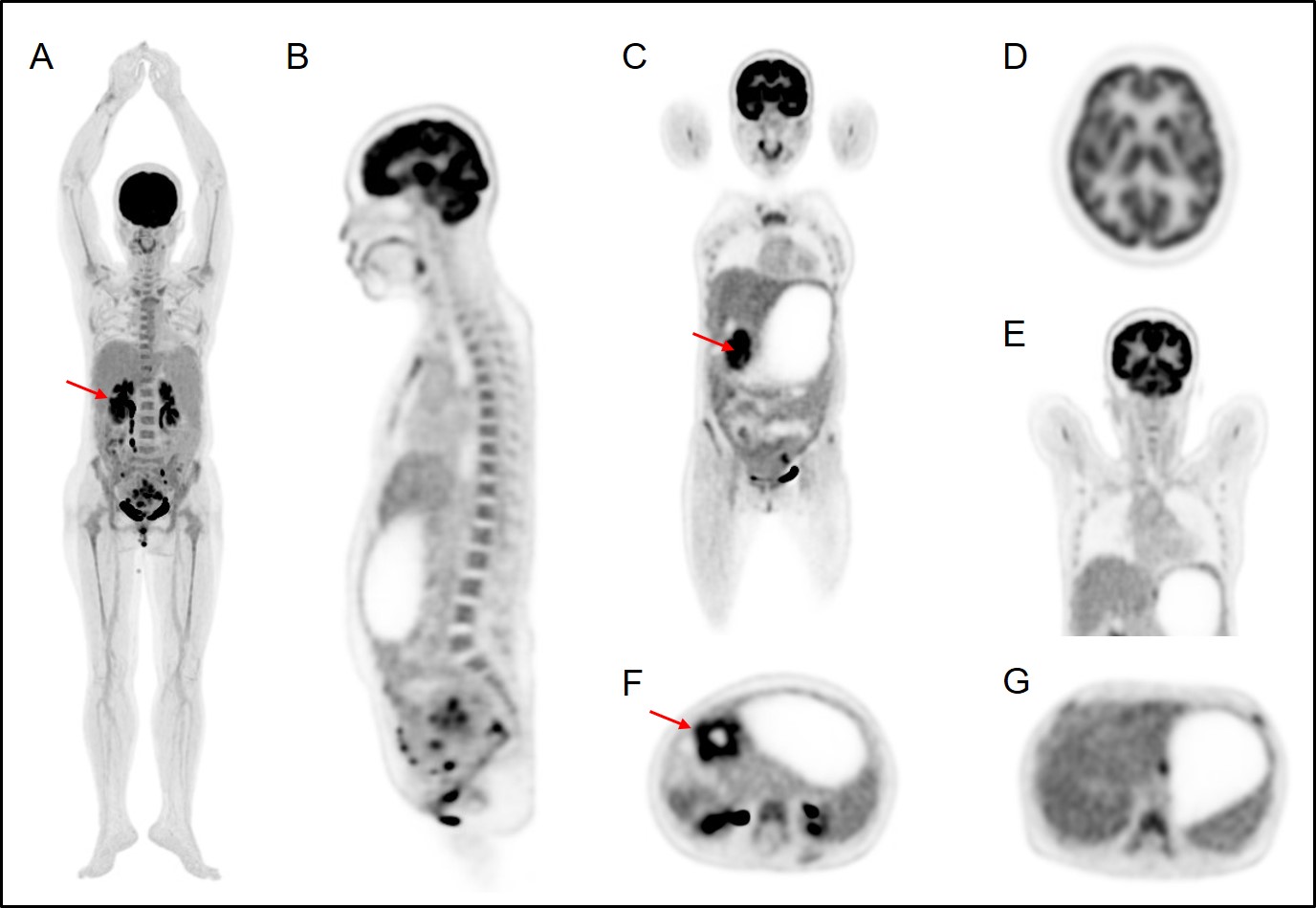
Supplementary Figure 6.** A case of 44-year-old female patient (53 kg, 156 cm) with colon cancer who underwent a PET/CT scan at 63-min post-injection of 53.28 MBq ^18^F-FDG (1.01 MBq/kg, 0.027 mCi/kg). The PET image was reconstructed with the suggested protocol of quarter-dose – the first 10-min acquisition time and 2 iterations.

*Pediatric full-dose examination.* A 6-year-old female pediatric patient (19.5 kg, 114 cm) with Burkitt lymphoma was intravenously injected with 69.93 MBq of ^18^F-FDG. A 10-min list-mode PET scan has been performed using uEXPLORER 64 min post-injection. PET images (Supplementary Fig. 7) were reconstructed with OSEM-TOF-PSF with the first 1-, 3-, and 10-min, 20 subsets, 2 iterations on a 3.125× 3.125×2.85 mm^3^ voxel grid. As for the maximum intensity projection (MIP) image, a trace of movement of the left hand were observed in a 10-min PET examination. In Supplementary Fig. 7, the transverse slice crossing the liver illustrated a false-positive suspected lesion at the stomach and a blurry body boundary due to movement. Fortunately, despite the motion artefacts in the 10-min PET images, the first 1-min PET images provide a clear visualization of the lymph nodes with clinically acceptable image quality.


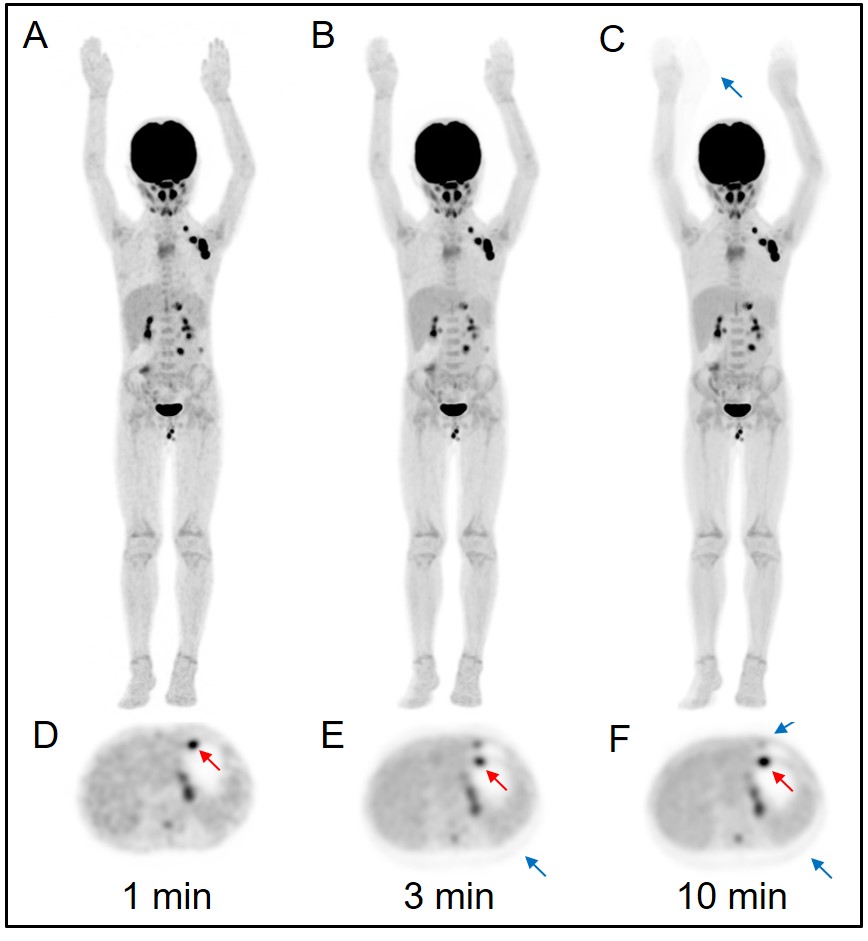

**Supplementary Figure 7.** A case of 6-year-old female pediatric patient with Burkitt lymphoma (19.5 kg, 114 cm) was intravenously injected with 69.93 MBq ^18^F-FDG (full-dose, 3.58 MBq/kg) and underwent a total-body PET/CT examination 64-min post-injection. Maximum intensity projection images (A-C), transverse images crossing the liver (D-F) were individually reconstructed with the acquisition time of the first 1-, 3-, and 10-min. The red arrows indicate the suspected lesion, and the blue arrows indicate the motion-induce false-positive lesions and trace of movement of body boundary. In the 10-min MIP (C), a trace of movement at hand appears.

*Phantom measurements in conventional short AFOV PET/CT scanner.* The measurement was carried out from a Siemens Biograph mCT PET/CT scanner that installed in our department. The scanner is with a bore diameter of 780 mm and with a AFOV of 22.1 cm. The NEMA phantom preparation, data acquisition, and data processing are same as previously stated but with following slight differences: (1) the tube voltage, tube current and the spiral pitch of the CT scan are 120 kV tube voltage, adaptive tube current exposure, and 0.8, respectively; (2) The same OSEM-TOF-PSF algorithm was utilized for reconstruction but with different matrix size of 200 ×200 (4.0728×4.0728 mm^2^) and 5 mm slice thickness (the FOV and the slice thickness is user unchangeable); (3) Image reconstruction iteration numbers was tuned from 2 to 5 instead of from 2 to 15 since there have no such choice in the work station. The Supplementary Figure 8 depicted the schematic diagram of duration-iterations-dependent PET image of NEMA IQ phantom with different injected activities from the transverse view for mCT scanner. And Supplementary Figure 9 demonstrates the iteration-and-duration-dependent CNR for different injected activity that from mCT scanner.


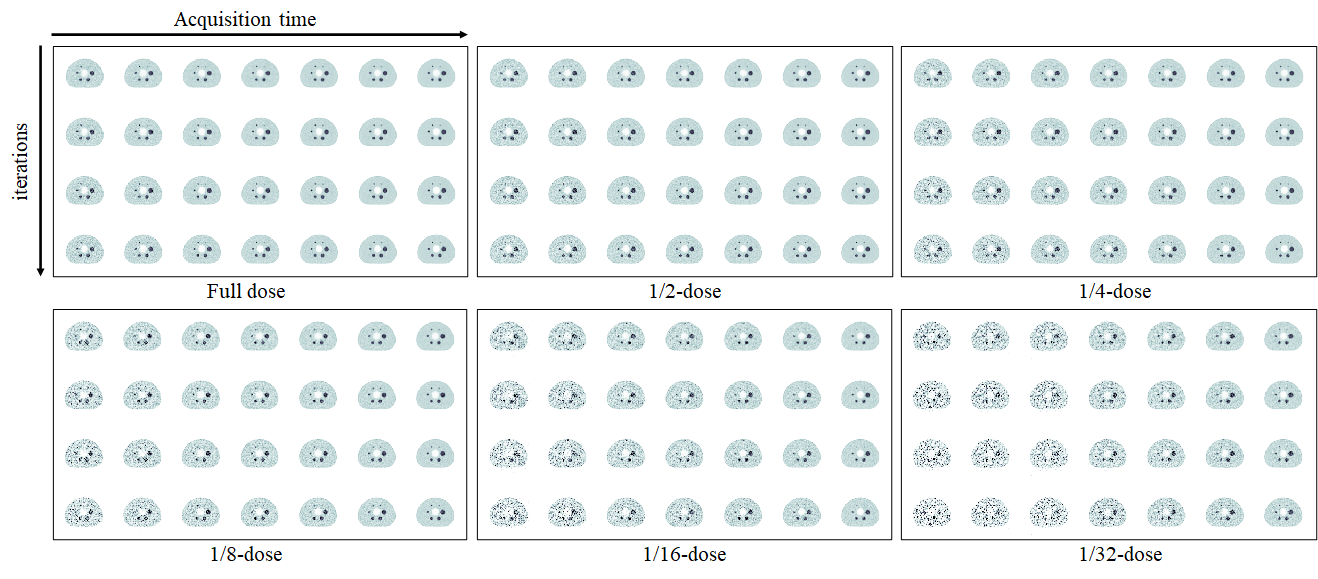


**Supplementary Figure 8.** The schematic figure of transverse image slices of the NEMA IQ phantom achieved at the different acquisition time, iteration number, and injected activity for conventional short AFOV scanner. The list-mode PET raw-data was truncated and respectively reconstructed into 40 s, 1 min, 2 min, 3 min, 5 min, 10 min and 30 min (ordered from left to right column), where the iteration number applied in OSEM-TOF-PSF reconstruction process were 2, 3, 4, 5 from top to bottom line, respectively.


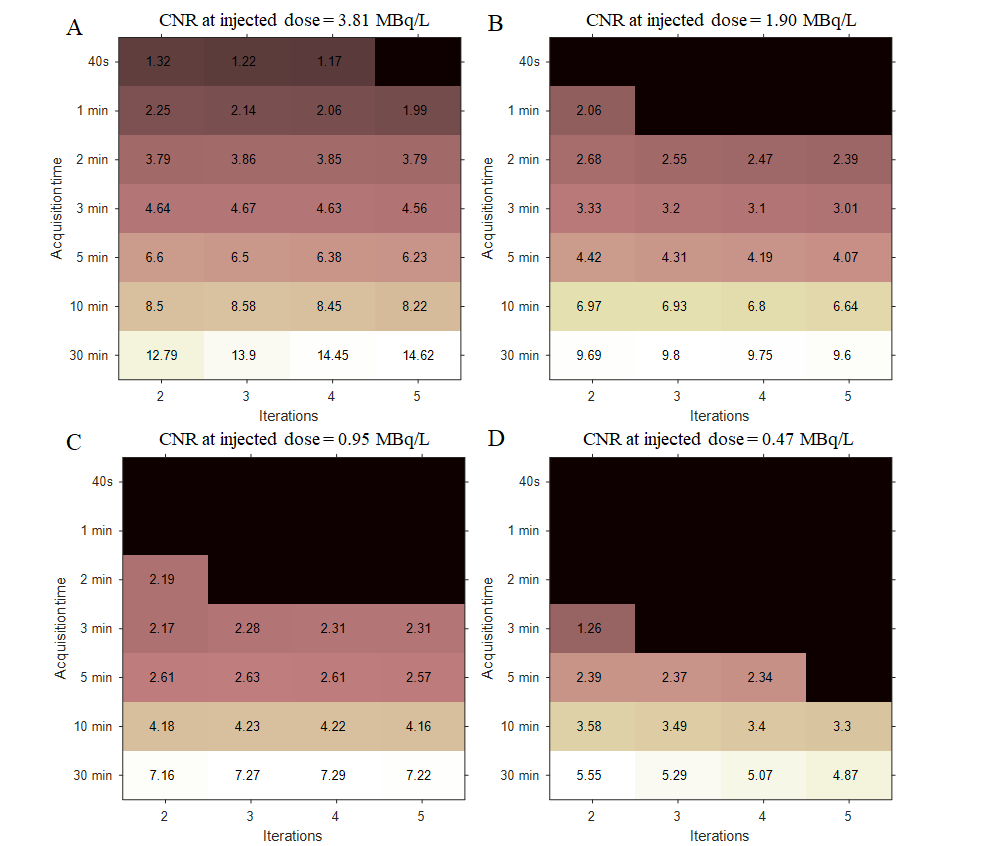


**Supplementary Figure 9.** Contrast-to-noise ratio (CNR) map derived from conventional short AFOV scanner. The dark area represents the corresponding BV value that is more than 15%.
